# Expression and Pharmacology of Endogenous Ca_v_ Channels in SH-SY5Y Human Neuroblastoma Cells

**DOI:** 10.1371/journal.pone.0059293

**Published:** 2013-03-25

**Authors:** Silmara R. Sousa, Irina Vetter, Lotten Ragnarsson, Richard J. Lewis

**Affiliations:** Institute for Molecular Bioscience, The University of Queensland, St. Lucia, Australia; University of Houston, United States of America

## Abstract

SH-SY5Y human neuroblastoma cells provide a useful *in vitro* model to study the mechanisms underlying neurotransmission and nociception. These cells are derived from human sympathetic neuronal tissue and thus, express a number of the Ca_v_ channel subtypes essential for regulation of important physiological functions, such as heart contraction and nociception, including the clinically validated pain target Ca_v_2.2. We have detected mRNA transcripts for a range of endogenous expressed subtypes Ca_v_1.3, Ca_v_2.2 (including two Ca_v_1.3, and three Ca_v_2.2 splice variant isoforms) and Ca_v_3.1 in SH-SY5Y cells; as well as Ca_v_ auxiliary subunits α_2_δ_1–3_, β_1_, β_3_, β_4_, γ_1_, γ_4–5_, and γ_7_. Both high- and low-voltage activated Ca_v_ channels generated calcium signals in SH-SY5Y cells. Pharmacological characterisation using ω-conotoxins CVID and MVIIA revealed significantly (∼ 10-fold) higher affinity at human versus rat Ca_v_2.2, while GVIA, which interacts with Ca_v_2.2 through a distinct pharmacophore had similar affinity for both species. CVID, GVIA and MVIIA affinity was higher for SH-SY5Y membranes vs whole cells in the binding assays and functional assays, suggesting auxiliary subunits expressed endogenously in native systems can strongly influence Ca_v_2.2 channels pharmacology. These results may have implications for strategies used to identify therapeutic leads at Ca_v_2.2 channels.

## Introduction

Voltage-gated Ca^2+^ channels (Ca_v_) are membrane proteins essential for the control of calcium signaling events, such as muscle contraction, gene expression, and neurotransmitter and hormone release. Dysfunction of Ca_v_ channels is related to a variety of heart, circulatory and neurological diseases; including arrhythmias, hypertension, some forms of epilepsy, migraine and other chronic diseases such as cancer, diabetes, ischemic brain injury and neuropathic pain [1], [2]. The Ca_v_ α subunit contains the voltage sensor and gating machinery and is the binding site for most inhibitors. This subunit comprises 4 domains each with six transmembrane segments. The pore is formed by the S5/S6 segments and the connecting pore loop, with channel opening gated by bending of the S6 segments at a hinge glycine or proline residue [3], [4]. The voltage sensor domain consists of the S1–S4 segments, with positively charged residues in S4 serving as gating charges [5] (for review see: [3], [6], [7]).

Based on the distinct pharmacological and electrophysiological properties of Ca_v_ channels, ten different gene subfamilies have been identified in vertebrates and classified as high voltage activated (HVA) Ca_v_1.1–4 (L-type), Ca_v_2.1 (P/Q-type), Ca_v_2.2 (N-type), Ca_v_2.3 (R-type); and low voltage activated (LVA) Ca_v_3.1–3 (T-type). The α subunit includes channels containing α_1S_, α_1C_, α_1D_, and α_1F_, which mediate L-type Ca^2+^ currents. The Ca_v_2 subfamily (Ca_v_2.1 to Ca_v_2.3) includes α subunits α_1A_, α_1B_, and α_1E_, which mediate P/Q-type, N-type, and R-type Ca^2+^ currents, respectively. The Ca_v_3 subfamily (Ca_v_3.1 to Ca_v_3.3) includes α subunits α_1G_, α_1H_, and α_1L_, which mediate T-type Ca^2+^ currents (**Table 1**) (for reviews see: [3], [8], [9]). Of these, Ca_v_2.2 has been of particular interest as a therapeutic target given the central role it plays mediating neurotransmitter release in nociceptive pathways such as presynaptic nerve terminals and dendrites [10]. Ca_v_ α subunits are co-expressed in native systems together with two or three auxiliary subunits (β, α_2_δ and γ), which undergo alternative splicing (for review see: [9]) and dramatically influence Ca_v_ channel function, intracellular trafficking and posttranslational modifications [11]. Indeed, when expressed alone in recombinant system, the α_1B_ subunit, for example, encodes a voltage-dependent calcium channel with kinetic properties different from those of native Ca_v_2.2 channels [9], [12]. In contrast, when co-expressed with auxiliary β and α_2_δ, increased current amplitudes are observed and the kinetics of activation and inactivation are closer to those of native channels [12].

**Table 1:** Primers used to identify Ca_v_ channels α subunits in SH-SY5Y cells.

Subtype	Accession Number	Primer Forward/Reverse	Size (bp)	Annealing T (°C)
Ca_v_1.1	NM_000069.2	CGCATCGTCAATGCCACCTGGTTTA/AGCACATTGTCGAAGTGGAAGTCGC	623	(?) ND
Ca_v_1.2	[19]	CTGCAGGTGATGATGAGGTC/GCGGTGTTGTTGGCGTTGTT	502	58 [19]
Ca_v_1.3	EU 363339.1	ACCCCCACCTGTAGGATCTCTCTCC/TCCTGACACTAGTCGAAGTGGTCGC	541	68
Ca_v_1.3	NM_001128840.1	GCTGCTGTGGAAGTCTCTGTCAAGC/TCAGTGATTCCACCACACACCACGA	343	68
Ca_v_1.4	NM_005183.2	AGGGACCCCTAAGCGAAGAAACCAG/ACCCCATGGCATCTTGCATCCAGTA	899	(?) ND
Ca_v_2.1	FJ040507.1	AGGACGAGGACAGTGATGAA/GCAGAGGAAGATGAAGGA AA	365	(?) ND
Ca_v_2.2	NM_000718.2	GGAACTGACTTCGACCTGCGAACAC/CCTCCTCTGCGTGGATCAGGTCATT	754	60
α_1B_Δ_1_	Bp 22+34	AGGAGATGGAAGAAGCAGCCAATCA/CCTTTCTGGTGTTTCATCTGGTGCA	900	58
α_1B_Δ_1_	Bp 23+33 [16]	CCAGAGGATGCAGACAATCAGCGGA/GCATCTTCTACCTGTCGAGGTACGC	900	60
α_1B_Δ_2_	Bp 21+31	CAGCCAATCAGAAGCTTGCTCTGCAAAAG/CTTTCGTTTGCGGTGGTCCCGCGGT	700	(65)
α_1B_Δ_2_	Bp 24+33 [16]	CAAGGATGAAGAGGAGATGGAAGAA/GCGTACCTCGACAGGTAGAAGATGC	1300	(?) ND
Ca_v_3.1	BC110995.1	GCTGCTGGAGACACAGAGTACAGGT/CTCGTGGTATTCGATGCCCATGCTG	397	60
Ca_v_3.2	NM_021098.2	CCTGATCCCTACGAGAAGATCCCGC/CACGGCTGAAGTACTTGCTGTCCAC	433	60
Ca_v_3.3	AF393329.1	AGATGCCCTTCATCTGCTCCCTGTC**/**AAGATCTCCTCGTAGCAGTCGCCAG	526	60

(?) ND: isoform not detected, unknown annealing temperature.

Cell-based systems are desirable in the field of high-throughput screening assays due to their similarity to *in vivo* environment. SH-SY5Y human neuroblastoma cells are derived from human sympathetic neuronal tissue. This cell line maintains in culture many of the properties of nerve cells, providing a useful model for the characterisation of molecules affecting human neuronal function, including endogenously expressed Ca_v_ channels [13]–[15]. In particular, SH-SY5Y cells have been an attractive model system for the study of Ca_v_2.2 function [13]. Although heterologous expression models provide control of subunit expression, native systems provide potentially more complex models which, when characterized, can help to determine the pharmacology of drugs in a native context and the physiology and pathophysiology of endogenously expressed receptors and channels. However, little is known about the Ca_v_ subtypes and auxiliary subunits endogenously expressed in SH-SY5Y cells, limiting the interpretation of pharmacological data. Here we report a detailed characterisation of endogenously expressed Ca_v_ channels expressed in SH-SY5Y cells using PCR and pharmacological approaches, with particular emphasis on the nociceptive target Ca_v_2.2.

## Results

### SH-SY5Y Cells Endogenously Express Multiple Ca_v_ Subtypes, Ca_v_2.2 Isoforms and Auxiliary Subunits

**Figure 1:**
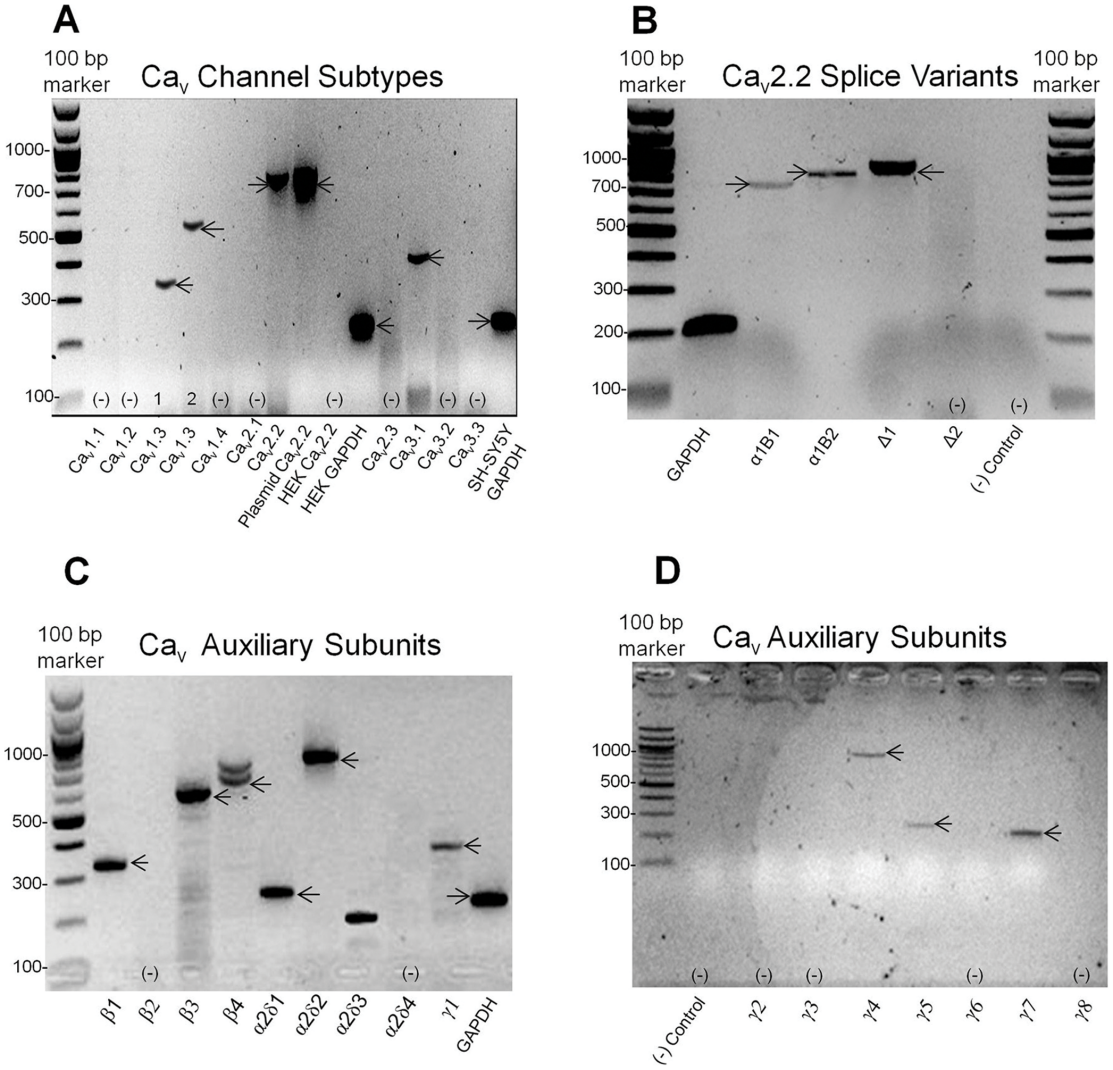
RT-PCR to identify the Ca_v_α and auxiliary subunit isoforms expressed in SH-SY5Y cells. Expression of Ca_v_α subtypes, auxiliary β, α_2_δ and γ subunits, as well as Ca_v_2.2 splice variant isoforms were determined in SH-SY5Y cells using standard RT-PCR and specific primers for each isoform. (**A**) SH-SY5Y cells endogenously express Ca_v_1.3 isoform 1, Ca_v_1.3 isoform 2, Ca_v_2.2 and Ca_v_3.1, but not Ca_v_1.1 and Ca_v_1.2, Ca_v_1.4, Ca_v_2.3, Ca_v_3.2 and Ca_v_3.3. Expected band sizes were (bp): Ca_v_1.3 isoform 1, 541; Ca_v_1.3 isoform 2, 343; Ca_v_2.2, 754; and Ca_v_3.1, 397, as indicated with arrows (**B**) SH-SY5Y cells endogenously express different Ca_v_2.2, α_1B_ splice variant isoforms. Bands with predicted sizes were (bp): α_1B1_, 728; α_1B2_, 854; Δ_1_, 900 bp. No band was detected for splice Δ_2_. (**C–D**) SH-SY5Y cells express the auxiliary β_1_, β_3_, and β_4_ but not β_2_; in addition to α_2_δ_1–3_, but not α_2_δ_4;_ and γ_1_, γ_4–5_ and γ_7_ but not γ_2–3_ and γ_8_ subunits. Expected band sizes were (bp, base pairs): β_1_, 331; β_3_, 594; β_4_, 731; α_2_δ_1_, 252; α_2_δ_2_, 878; α_2_δ_3_, 132 and γ_1_, 367; γ_4_, 909; γ_5_, 257; and γ_7_, 910.

We assessed expression of mRNA transcripts for Ca_v_ subtypes and auxiliary α_2_δ, β and γ subunits isoforms in SH-SY5Y cells by performing RT-PCR using specific primers (**Fig. 1A–D**). Bands with the predicted sizes were detected for Ca_v_1.3, Ca_v_2.2, and Ca_v_3.1, while Ca_v_1.1, 1.2, 1.4, 2.1, 2.3, 3.2 and 3.3 were not detected (**Fig. 1A**). In addition, bands of expected sizes for (**Table 2**) β_1_, β_3_, β_4_, α_2_δ_1–3_, γ_1_, γ_4_, γ_5_ and γ_7_ auxiliary subunits (**Fig. 1C**–**D**) were also identified. Since splice variants can be generated by alternative RNA processing, which can influence function and pharmacology [16], we also investigated the expression of some human splice variants [16], [17]. PCR bands with the predicted sizes for Ca_v_1.3 isoforms 1 and 2; full length Ca_v_2.2, α_1B1_ (Gene bank accession number M94172.1), shorter α_1B_ variant, α_1B2_ (Gene bank accession number M94173.1) [17]; and Δ_1_ (but not Δ_2_) [16], [18] were detected for the first time in the SH-SY5Y cells (**Fig. 1A** **,** **Table 1**).

**Table 2:** Primers used to identify Ca_v_ channel auxiliary subunits in SH-SY5Y cells.

Subunit	Accession Number	Primer Forward/Reverse	Size (bp)	Annealing T (°C)
β_1_	NM_000723.3	ATGCACGAGTACCCAGGGGAG/CAGCGCAGTAGCGGGCCTTATT	331	60
β_2_	NM_000724.3	TCGCTTGCCAAACGCTCGGT/ATGACGGCTGCGCTGCTTGT	909	(?) ND
β_3_	NM_000725.2	GCAGCAGCTCGAAAGGGCCA/ATGCTGGAGCGGGCAGAGGA	594	65
β_4_	NM_001005747.2	TGAAGACTCGGAGGCTGGTTCAGC/TGGACCGGGTGTTCGAACGT	731	(?) ND
α_2_δ_1_	NM_000722.2	TGCTCATCGGCCCCTCGTCG/CCAGGCGCACCAGGGCTTTAG	252	60
α_2_δ_2_	NM_001174051.1	AGCCTAGGCAGGCGCACACT/TCTGCACTAGCTCACACTGCTCCGG	878	60
α_2_δ_3_	NM_018398.2	GGACGAGAGGCTGCGTTTGCA/GGGCCGGCTAAGCACGTGAA	132	65
α_2_δ_4_	NM_172364.4	TGGCCTGGGCCTTTGTGCAG/GCCTCCTCGGCAGCTTCCAC	328	(?) ND
γ_1_	NM_000727.3	TGCTGGCCATGACAGCCGTG/AACATGGACGCGGGTCGCAG	367	60
γ_2_	NM_006078.3	TCTCTGGGCCTTAATTTTCCCC/TTTTCACAGACCCCCAAAGACA	439	(?) ND
γ_3_	NM_006539.3	CTCCCCTTCCCCTTTCCTTAAC/AGCTGGGATTTCCTTTCTGGAG	840	(?) ND
γ_4_	NM_014405.3	TTTGCACGAAGGTTGTGCTG/TTGCTCTCCTGGCGTTGATT	909	62–64
γ_5_	NM_145811.2	GATCAAGATGTCCCTGCACTCA/CAGAGACAAAGGCCAGTATCGT	257	64
γ_6_	NM_145814.1	TGCTCAGTAAAGGTGCAGAGTT/CTCGGTGGTTGCTTAGAGAAGT	334	(?) ND
γ_7_	NM_031896.4	ACTGGCTGTACATGGAAGAAGG/TGAAATAAGGGAGTCTGTGGGC	910	65
γ_8_	NM_031895.5	TGCTGAAGCATAGTCATGGTGT/CCTCTGCCTTCTCAGTGAACTT	987	(?) ND

(?) ND: isoform not detected, unknown annealing temperature.

The best annealing temperature for each gene analysed (see **Table 1** **–** **2**) was determined using a gradient PCR protocol in rounds of control experiments prior to testing each Ca_v_ gene-specific primer. Target-specific primers for the housekeeping gene GAPDH were designed as previously described [19] and GAPDH was detected in all PCRs, indicating amplifications were cDNA specific. PCR master mix using random primers without cDNA was used as negative gDNA control in all PCRs. Specificity of primers was demonstrated in a range of control experiments (data not shown), including detection of Ca_v_2.2 plasmid but not other Ca_v_ subtypes by Ca_v_2.2 primers; and absence of Ca_v_2.2 in HEK cells. β_1_ and α_2_δ_1_ primers were positive for β_1_ and α_2_δ_1_ plasmids, while the same primers were negative for β_2–4_ and α_2_δ_2–4_ (data not shown), indicating primers were selective for β_1_ and α_2_δ_1_ auxiliary subunits. The identity of each of these PCR products, including γ_1_, γ_4_, γ_5_ and γ_7_, was confirmed by sequencing analysis (data not shown).

### Displacement of ^125^I-GVIA Binding from SH-SY5Y Cell Membranes

**Figure 2:**
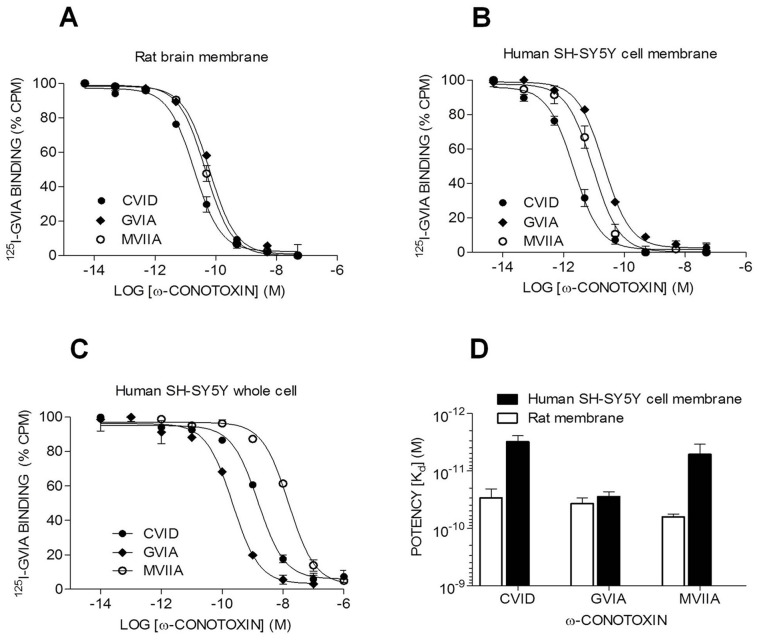
Displacement of ^125^I-GVIA from SH-SY5Y whole cell and membranes byω-conotoxins. Displacement of ^125^I-GVIA binding to Ca_v_2.2 expressed in rat brain and SH-SY5Y intact/whole cell and membranes. (**A**) Displacement of^ 125^I-GVIA from rat brain membranes. (**B**) Displacement of^ 125^I-GVIA from human SH-SY5Y cell membranes. (**C**) Displacement of^ 125^I-GVIA from human SH-SY5Y whole cell. (**D**) ω-Conotoxins affinity (K_d_ ± SEM) to displace ^125^I-GVIA from rat brain membranes and human SH-SY5Y cell membranes. Data are mean ± SEM of triplicate data from a representative experiment best fitted to a single-site competition model using GraphPad Prism.

GVIA is a highly selective Ca_v_2.2 blocker [20] and ^125^I-GVIA binding assays have been well established using rat brain membranes [21]–[23]. We performed binding assays and confirmed SH-SY5Y cells contain ^125^I-GVIA binding sites which can be fully displaced by Ca_v_2.2 selective inhibitors ω-conotoxins CVID, GVIA and MVIIA. Affinities of ω-conotoxins for human and rat Ca_v_2.2 channels were next compared using these assays. CVID, GVIA and MVIIA each fully displaced ^125^I-GVIA binding to crude rat brain membranes with similar affinities (pIC_50_± SEM values; CVID 10.53±0.15, GVIA 10.43±0.16, and MVIIA 10.19±0.04) (**Fig. 2A** **,** **Table 3**), consistent with earlier studies [21]. Intriguingly, the affinity of GVIA (pIC_50_ 10.55±0.15) to displace ^125^I-GVIA binding to SH-SY5Y membranes was similar to that shown in rat brain, while both CVID and MVIIA had significant higher affinity for the human cell membranes (pIC_50_s of 11.51±0.12 and 11.29±0.23, respectively) than for rat brain membranes (**Fig. 2A, B and D** **,** **Table 3**). In addition, the affinities of these ω-conotoxins dramatically decreased when determined on the intact SH-SY5Y cells instead of membranes, with GVIA affinity shifted ∼10-fold, and CVID and MVIIA affinity shifted ∼100-fold (see **Fig. 2C** **,** **Table 3**).

**Table 3:** ω-Conotoxin affinities (IC_50_± SEM) to displace ^125^I-GVIA binding.

ω-Conotoxin	Rat membrane K_d_ (nM)	SH-SY5Y membrane K_d_ (nM)	Whole SH-SY5Y K_d_ (nM)
**CVID**	0.034±0.013	0.0034±0.009	3.2±0.2
**GVIA**	0.043±0.013	0.033±0.012	0.27±0.084
**MVIIA**	0.064±0.007	0.0065±0.0019	10±0.085

### Pharmacology of the Endogenously Expressed Ca_v_ Channels

**Figure 3:**
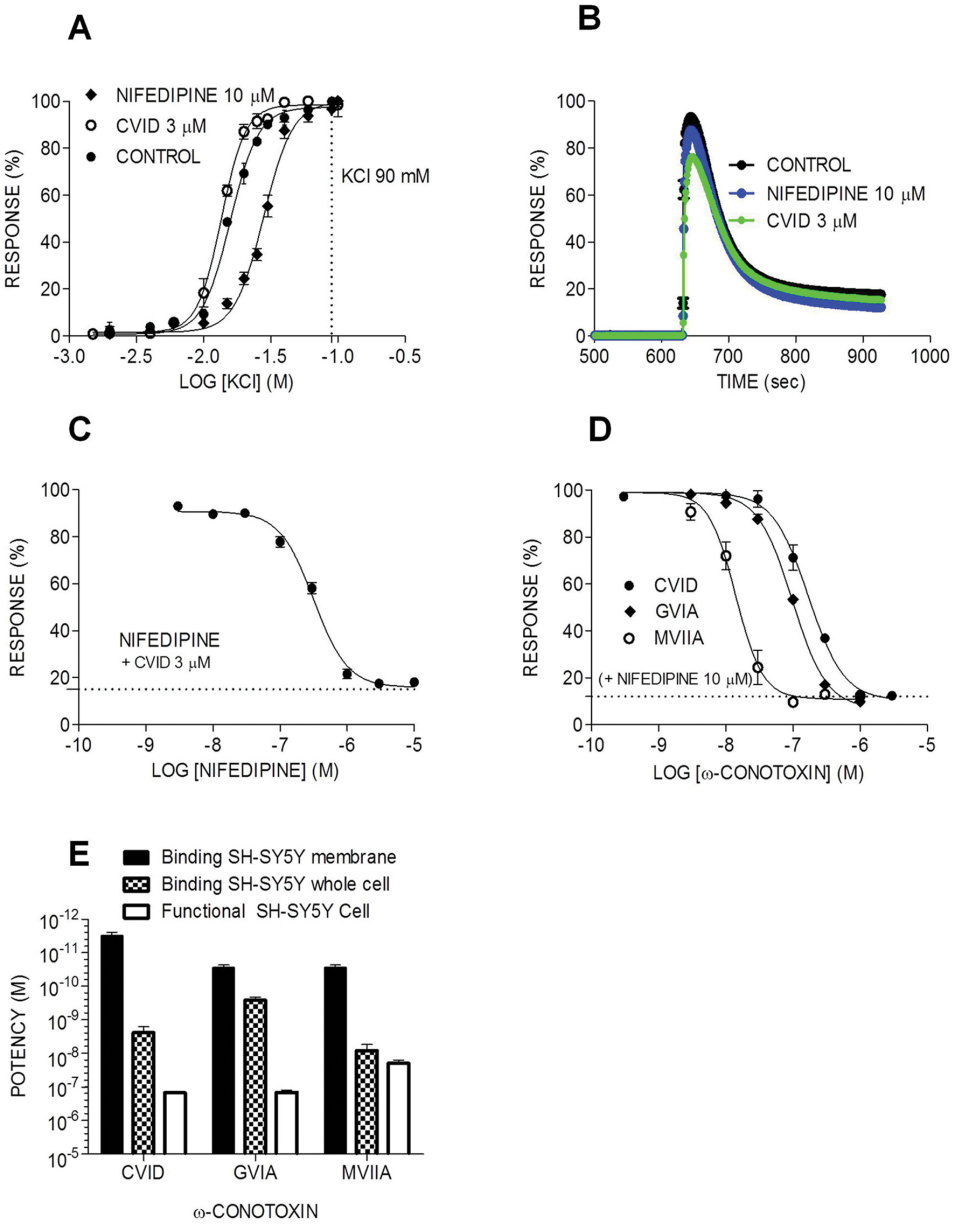
Ca_v_2.2 and Ca_v_1 channels endogenously expressed in SH-SY5Y cells are functional. Data obtained from fluorescent Ca^2+^ imaging assays of KCl-evoked Ca^2+^ responses in SH-SY5Y cells. **(A)** Ca_v_1 and Ca_v_2.2 activation in the presence of CVID (open ball) and nifedipine (filled ball), respectively, shifted control KCl-evoked Ca^2+^ responses (quadrilateral) significantly in SH-SY5Y cells (p>0.05). (**B**) Time course of Ca^2+^ responses is shown for control KCl 90 mM (black), KCl in the presence of nifedipine (blue) and KCl in the presence of CVID (green). (**C**) Concentration-response curve for nifedipine inhibition of Ca_v_1 responses (**D**) Concentration-response curves for CVID, GVIA and MVIIA inhibition of Ca_v_2.2 responses. The responses were normalized using controls: positive KCl and negative PSS buffer; and plotted across increasing concentrations of antagonists (**E**) Comparison of ω-conotoxins CVID, GVIA and MVIIA potencies (IC_50_/K_d_ ± SEM of n = 3–4 replicates for each experiment, n = 3 experiments) in displacing ^125^I-GVIA from SH-SY5Y whole cell and SH-SY5Y cell membranes with the functional assays data.

To investigate if the Ca_v_ channels endogenously expressed in SH-SY5Y cells were functional, and to further study the pharmacology of these channels, we assessed KCl-evoked Ca_v_ responses using a fluorescent high-throughput Ca^2+^ imaging assay on the FLIPR^Tetra^ (Fluorescent plate image reader, Molecular Devices, Sunnyvale, CA) (**Fig. 3A–D**). CaCl_2_ (5 mM) was added to the KCl stimulation solution in all experiments to maximize the Ca^2+^ influx signal. Co-addition of 90 mM KCl and 5 mM CaCl_2_ evoked a large transient response indicating increase in intracellular Ca^2+^ (**Fig. 3A–B**). Concentration-response curves for KCl-mediated stimulation showed activation of Ca_v_ responses with an EC_50_ of 17.3 mM (pEC_50_ 1.88±0.06, Hill slope of 2.5) (**Fig. 3A** **,** **Table 4**), similar to previously described values [24]–[26]. To assess the contribution of each Ca_v_ channel expressed in SH-SY5Y cells to the KCl-evoked Ca^2+^ responses, we determined concentration-response curves for KCl/Ca^2+^ stimulation in the presence of subtype-specific inhibitors. The Ca_v_1 (L-type) inhibitor nifedipine was used at a concentration (10 µM) that does not affect responses of other Ca_v_ subtypes (N, R, P/Q or T-type) [27], to isolate non-L-type responses. The KCl concentration-response curve was shifted to the right in the presence of nifedipine (EC_50_ of 20.4 mM, pEC_50_ 1.69±0.12, Hill slope of 2.9) (**Fig. 3A** **Table 4**). Conversely, the Ca_v_2.2 (N-Type) inhibitor ω-conotoxin CVID was used at a concentration that does not affect responses of other Ca_v_s (up to 3 µM) [21], [28], to isolate non-N-type responses. Compared to responses in the presence of nifedipine, the KCl concentration-response curve was shifted to the left in the presence of CVID (EC_50_ of 18.6 mM, pEC_50_ 1.86±0.10, Hill slope of 3.5) (**Fig. 3A** **,** **Table 4**). These differences can be accounted for by the electrophysiological properties of each Ca_v_ channel subtype identified, since L-type requires a larger depolarization than N-type to be activated [28], and the control KCl responses is a result of activation of both channel types. These results confirm that SH-SY5Y cells express functional Ca_v_ subtypes, including Ca_v_1 and Ca_v_2.2, which can be pharmacologically isolated using selective inhibitors. The observed pharmacology is consistent with the subtypes identified in our PCR experiments and with previous reported electrophysiological data [14], [15].

**Table 4:** Potency (IC_50_± SEM) of Ca_v_ channel modulators on functional assays.

Ca_v_ Activator/Inhibitor	Ca^2+^ Stimulation EC_50_ (mM)	Ca^2+^ Inhibition IC_50_ (µM)
KCl	17.28±3.41	−
KCl+CVID	18.61±3.22	−
KCl+NIFEDIPINE	20.35±3.17	−
CVID	−	0.16±0.025
GVIA	−	0.15±0.09
MVIIA	−	0.024±0.005
NIFEDIPINE	−	0.23±0.046
MIBEFRADIL	−	3.0±0.031
PIMOZIDE	−	1.3±0.097
ω-AGATOXIN TK	−	NDR
SNX 482	−	NDR

NDR: Non-detectable response.

Since 90 mM KCl/5 mM CaCl_2_ elicits maximal Ca_v_1 and Ca_v_2.2 responses (**Fig. 3A** **)**, we used this combination to further characterize the Ca_v_ channel subtypes expressed in SH-SY5Y cells. Concentration-response curves for nifedipine at Ca_v_1 channels were generated in the presence of saturating concentration of CVID (3 µM). Under these conditions, nifedipine inhibited KCl evoked Ca^2+^ responses with an IC_50_ of 0.28 µM (pIC_50_ 6.5±0.052) (**Fig. 3C** **,** **Table 4**), consistent with reports for nifedipine block of L-type responses in neuronal cells [29]. To characterize Ca_v_2.2 pharmacology, inhibition by ω-conotoxins was determined in the presence of a near saturating concentration of nifedipine (10 µM). Under these conditions, the potency of CVID was IC_50_ 0.16 µM (pIC_50_ 6.87±0.078), GVIA 0.15 µM (pIC_50_ 6.84±0.06) and MVIIA 0.024 µM (pIC_50_ 7.7±0.13) (**Fig. 3D** **,** **Table 4**). These results are consistent with previous studies on MVIIA [22]–[24], [26] and CVID [22]–[24] inhibition of N-type responses in native and recombinant systems, when Ca_v_2.2 was co-expressed with β and α_2_δ subunits [22]–[24]. In contrast, GVIA potency at Ca_v_2.2 expressed in SH-SY5Y cells (IC_50_ of 0.15 µM; pIC_50_ 6.8±0.072) was consistently lower than previously described for heterologous expressed rat [24], [26] and human [25] α_1B_ co-expressed with α_2_δ_1_ and β_3_, but similar to data obtained using native expression systems such as dissociated rat DRG cells [30] and chicken synaptosomes [31].

**Figure 4:**
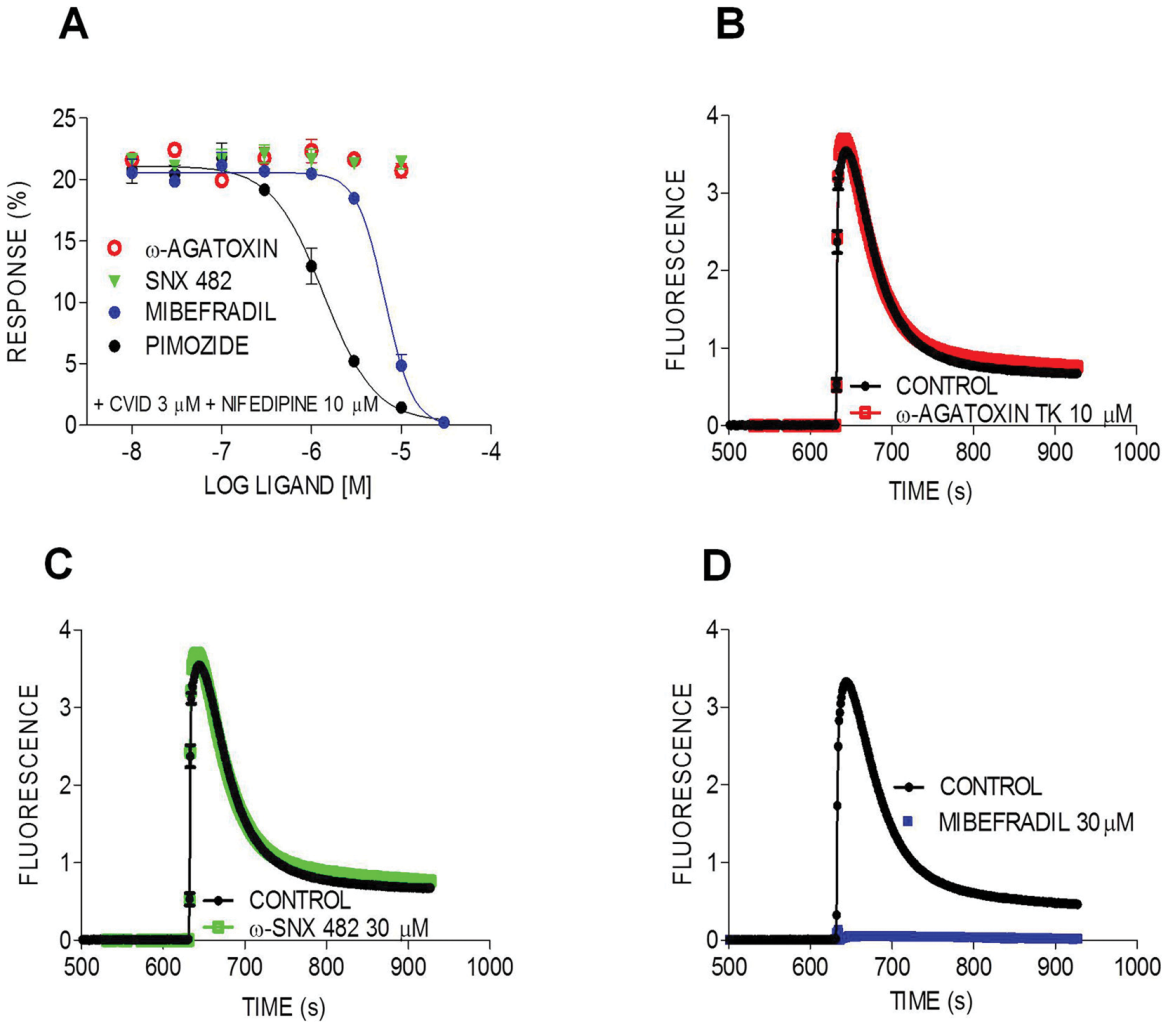
Characterization of resistant Ca^2+^ responses in SH-SY5Y cells. Data obtained from fluorescent Ca^2+^ imaging of KCl-evoked Ca^2+^ responses in SH-SY5Y cells. (**A**) Concentration-response curves for mibefradil, pimozide, ω-agatoxin TK and SNX 482 in inhibiting resistant KCl-evoked Ca^2+^ responses in SH-SY5Y cells, pretreated with CVID (3 µM) plus nifedipine (10 µM) **(B–D)** Time course of transient Ca^2+^ responses activated by 90 mM KCl/5 mM CaCl_2_, in the presence of CVID (3 µM) and nifedipine (10 µM) and following the addition of agatoxin TK, SNX-482 and mibefradil.

A small portion (5–15%) of the KCl-evoked responses was insensitive to block by co-application of 10 µM nifedipine and 3 µM CVID (**Fig.3C–D**). To pharmacologically characterize these remaining responses, we assessed the effects of Ca_v_2.1 and Ca_v_2.3 subtype-specific inhibitors, as well as of compounds with activity at Ca_v_3, on the Ca^2+^ responses evoked by 90 mM KCl/5 mM CaCl_2_, in the presence of both CVID and nifedipine. The Ca_v_2.1 blockers ω-agatoxin IVA (data not shown) and ω-agatoxin TK did not significantly affect KCl-evoked Ca^2+^ responses at concentrations up to 10****µM (**Fig. 4A–B** **,** **Table 4**). The Ca_v_2.3 antagonist SNX 482 also had no significant inhibitory effect at concentrations up to 10****µM (**Fig. 4A and C** **,** **Table 4**). On the other hand, mibefradil (30 µM), a benzimidazolyl-substituted tetraline reported to inhibit Ca_v_3 responses in different systems with weak affinity [32], [33] fully inhibited these remaining responses with an IC_50_ of 3 µM (pIC_50_ 5.3±0.035) (**Fig. 4A and D** **,** **Table 4**). Similar IC_50_ values for mibefradil block of T-type responses in native systems have been previously reported (see [32]–[35]). In addition, another Ca_v_3 inhibitor, the antipsychotic pimozide, also fully inhibited the remaining responses with an IC_50_ of 1.3 µM (pIC_50_ 5.2±0.097) (**Fig. 4A** **,** **Table 4**), similar to previously reported literature values [34]. These findings are in agreement with our PCR (**Fig. 1A–B**) which detected mRNA transcripts for Ca_v_3.1, but neither Ca_v_2.1 nor Ca_v_2.3 was identified.

## Discussion

Ca_v_2.2 channels play a key role in regulating nociception. Inhibition of Ca_v_2.2 at the spinal cord produces analgesia in animal models of pain [23], [36] and in humans [37], with direct (eg. Prialt) and indirect (eg. gabapentin) inhibitors among some of the most recently developed analgesics [18]. Neuroblastoma cells, including the sympathetically derived human neuroblastoma cell line SH-SY5Y, provide excellent model systems to study Ca_v_2.2 channels in a native context [14], [15]. However little is known about the Ca_v_α and auxiliary subunits expressed, limiting interpretation of pharmacological data from these cells. To address this limitation, we have characterized the expression and pharmacology of Ca_v_ channels in SH-SY5Y cells and investigated mechanisms likely to influence the pharmacology of ω-conotoxins at Ca_v_2.2 channels.

Previous electrophysiological studies have identified L- and N- currents from high voltage activated channels Ca_v_1 and Ca_v_2.2 in SH-SY5Y cells, but not low voltage activated T- type currents from Ca_v_3 channels [13]–[15], [38]. In contrast, we detected mRNA transcripts for the N-type (Ca_v_2.2), two L-type (Ca_v_1.3 isoform 1 and 2) and one T-type isoform (Ca_v_3.1). In addition, we also detected mRNA transcripts for Ca_v_2.2 splice variants, including α_1B2_ (74 amino acid shorter) [16], [17], [39] and the splice α_1B_Δ_1_ (382 amino acid shorter) [16].

Functional Ca_v_ responses elicited by addition of KCl/CaCl_2_ were assessed using a fluorescent high-throughput Ca^2+^ imaging assay on the FLIPR^Tetra^. KCl has been used extensively to activate Ca_v_ responses in a diversity of functional assays ([24], [26], [40]). Addition of high concentrations of KCl causes a change in membrane potential, which in turn leads to opening of Ca_v_ channels, influx of Ca^2+^ and a resultant increase in intracellular fluorescence. While the change in membrane potential elicited by addition of KCl at the concentrations used here is approximately linear, accumulation of intracellular Ca^2+^ is saturable and fits a sigmoidal concentration-response curve because a change in membrane potential leads to a finite change in channel open probability and thus Ca^2+^ influx.

Ca_v_2.2 channels expressed in SH-SY5Y cells were functional and generated KCl activated responses that were inhibited by ω-conotoxins CVID, GVIA and MVIIA in the presence of saturating concentrations of nifedipine. As expected, when L-type responses were isolated by addition of saturating concentrations of CVID, nifedipine concentration-dependently blocked KCl responses. However, a small response remained (5–15%) in the presence of a combination of Ca_v_2.2 and Ca_v_1 inhibitors. This resistant response was completely abolished by the Ca_v_3 inhibitors mibefradil and pimozide. While mibefradil and pimozide are not specific inhibitors of T-type currents and also inhibit L-type channels [32], [41], inhibition of the residual Ca^2+^ response was also observed in the presence of saturating concentrations of nifedipine, suggesting that activity of these compounds at L-type channels did not contribute to inhibition of residual response. Based on our observations that this resistant response was not blocked by inhibitors of L-type (nifedipine), N-type (CVID), R-type (SNX 482) or P/Q-type channels (ω-agatoxin), but was completely abolished by compounds with known activity at T-type channels, it seems plausible that this response may be mediated by Ca_v_3.1, which mRNA expression was detected in SH-SY5Y. Alternatively, it is known that the Δ_1_ splice variant, which mRNA expression was detected in SH-SY5Y cells, is significantly more resistant to the blockade by MVIIA and GVIA [42]. While inhibition by CVID of the Ca_v_2.2 splice variants detected in SH-SY5Y cells has not been characterised, it is possible that, akin to inhibition of Na_v_ channels by the µ-conotoxin GIIIA, complete current inhibition by CVID cannot be achieved for these splice variants. Alternatively, the response remaining in the presence of nifedipine and CVID could represent another undefined resistant current, or an artifact of the KCl/Ca^2+^ activation buffer used in this study.

Development of non-electrophysiological HTS Ca_v_3 channel assays has been hampered by some of the properties of this channel, including their low voltage threshold for activation and inactivation and rapid inactivation kinetics. However, although T-type currents inactive rapidly, fluorescence Ca^2+^ assays detect accumulation of intracellular Ca^2+^ rather than currents, and are thus not subject to the same temporal resolution constraints. In addition, compared to heterologous systems, SH-SY5Y cells have a relatively hyperpolarised resting membrane potential [43], which would be conducive to channels being present in the resting state. Accordingly, Ca^2+^ assays at Ca_v_3 channels using the FLIPR have been successfully developed [40] and it is clearly conceivable that functional responses of Ca_v_3.1 expressed in SH-SY5Y cells could be elicited using KCl/Ca^2+^ stimulation.

In addition to functional characterization, we also confirmed Ca_v_2.2 expression at the protein level using^ 125^I-GVIA binding assays. The ω-conotoxins CVID, GVIA and MVIIA each fully displaced ^125^I-GVIA binding to SH-SY5Y cell membranes with high affinity. Interestingly, while the affinity of GVIA was not significantly different between species, CVID and MVIIA affinities were ∼10-fold higher in human SH-SY5Y membranes compared to rat brain membranes. These results support the findings that MVIIA and CVID interacts with Ca_v_2.2 human channels through a different pharmacophore, as compared with GVIA [44].

Variation in the affinity of ω-conotoxins between species is likely influenced by Ca_v_α splice variants, with differences in toxin sensitivity, time course and voltage-dependence of inactivation, single channels conductance, gating behavior and sensitivity to G-protein-mediated modulation reported for splice isoforms endogenously expressed in neuronal cells of rat, mouse, rabbit and humans 16,17,39,42,45–48 (for review see: [46]). In pain, the Ca_v_2.2 splice variant 37a replaces the usual variant 37b in a specific subset of nociceptive neurons, and thus may represent a potential therapeutic target [42], [46], [49]. However, this variant has to date only been described in rat dorsal root ganglion neurons, and is not known to be present in human tissue.

Additional human splice variants include two α_1B_ isoforms that have long or short C-termini [17], and two human forms that lack large parts of the domain II-III linker region, including the synaptic protein interaction site. These splice variants, termed Δ_1_ and Δ_2_, have been previously isolated from IMR32 human neuroblastoma cell line and human brain cDNA libraries [16]. We have identified mRNA transcripts for the full length α_1B1_, α_1B2_ (74 amino acid shorter) [16], [17], [39] and the splice variant Δ_1_ (382 amino acid shorter) [16] in SH-SY5Y cells. The α_1B1_ is an axonal/synaptic isoform, while α_1B2_ is restricted to neuronal soma and dendrites [39], [50], however, apart from differential susceptibility to Gαi/Gαo-versus Gαq-mediated inhibition, little is known regarding its biophysical and pharmacological properties. On the other hand, the Δ_1_ splice variant has lost part of the synaptic protein interaction (synprint) site and is thus unlikely to play a role in fast synaptic transmission, with shifts in the voltage dependence of steady-state inactivation and a more rapid recovery from inactivation compared to full length α_1B1_ [16]. Importantly and clinically relevant, Δ_1_ variant was significantly more resistant to the blockade by MVIIA and GVIA; however the degree of effect varied for each toxin [16]. Thus, expression of the Δ_1_ variant in SH-SY5Y cells may contribute to the reduced ω-conotoxin affinity observed. While expression of these splice variants in SH-SY5Y cells was detected using gene specific primers, which have been extensively validated in the literature [17], further confirmation of expression at the protein level is warranted.

Ca_v_ channel auxiliary subunits can also influence the pharmacology of Ca_v_ inhibitors, with ω-conotoxins displaying reduced affinity in the presence of the α_2_δ subunit [11], [22]–[24], [27], [51]. Specifically ω-conotoxins GVIA, MVIIA and CVID had reduced affinity when α_2_δ_1_ subunit was co-expressed with the Ca_v_ α_1B_ [23]. α_2_δ up-regulation has been associated with chronic pain and epilepsy, with gabapentin and pregalin binding to α_2_δ reducing Ca_v_2.2 trafficking and the symptoms of pain [11]. The α_2_δ_1_–_3_, β_1_, β_3_ and β_4_, γ_1_, γ_4–5_ and γ_7_ subunits were detected in SH-SY5Y cells and potentially contribute to the differences in ω-conotoxins potency in whole cell vs. membrane assays.

The γ_1_ subunit was originally identified in skeletal muscle in complex with Ca_v_1 channels [52], but effects of this subunit on the ω-conotoxins affinity at Ca_v_2.2 have not been determined. In contrast, co-expression of the γ_7_ subunit almost abolished the functional expression of Ca_V_2.2 in either *Xenopus oocytes* or COS-7 cells [53], [54]. The neuronal γ_2_ is associated with epileptic and ataxic phenotypes of stargazer mouse [55], but was not detected in SH-SY5Y cells. The γ_5_ and γ_7_ subunits represent a distinct subdivision of the γ subunit family of proteins identified by structural and sequence homology to stargazing. The γ_4_ subunit affected only the Ca_v_2.1 channel [55], [56]. The γ_5_ subunit may be a regulatory subunit of Ca_v_3.1 channels (for review see: [57]). These subunits may also potentially contribute to differences in ω-conotoxins binding affinities observed in whole cell vs. membrane assays.

While auxiliary subunits affect ω-conotoxin affinity in functional studies, this quaternary complex is likely to be disrupted upon preparation of homogenized membranes for the binding assays [58]. To examine this possibility, we studied the ability of GVIA to displace ^125^I-GVIA from whole SH-SY5Y cells compared to homogenized membranes. Interestingly, ω-conotoxins CVID, MVIIA and GVIA had higher affinity to displace ^125^I-GVIA from the homogenized membranes compared to the whole cells, an effect that was most pronounced for CVID and MVIIA (∼100-fold) compared to GVIA (∼10-fold). We have previously reported a similar trend for both CVID and MVIIA in heterologous expression system with and without the α_2_δ subunit [22]. Potency estimates obtained with the functional assays were significantly lower than estimates obtained in whole cell radioligand binding assays. The relatively high level of Ca^2+^ in the physiological saline used traditionally for functional assays compared to binding assays could contribute to these differences, since Ca^2+^ non-competitively inhibits ω-conotoxin binding [21]. However, our whole cell data was also obtained by incubating ω-conotoxins in a Ca^2+^-free physiological saline solution and the origin of these differences is unclear. Interestingly, this effect was most marked for GVIA, intermediate for CVID and insignificant for MVIIA.

In summary, we have characterized functional Ca_v_ channels expressed in SH-SY5Y human neuroblastoma cell line. Our studies have shown expression of different Ca_v_α splice variants, in conjunction with auxiliary subunits in a native context, can modulate the pharmacology of Ca_v_2.2 channel inhibitors. SH-SY5Y cell line provides a useful model for the investigation of novel human Ca_v_2.2 inhibitors and is amenable to the establishment of high-throughput assays [59], which can be adapted to detect endogenously expressed human Ca_v_1.3, Ca_v_2.2 and possibly Ca_v_3.1, in the presence of appropriate inhibitors. These assays are expected to prove useful for the discovery and pharmacological characterization of novel Ca_v_ channel modulators targeting human Ca_v_ related diseases.

## Materials and Methods

### Reverse Transcriptase Polymerase Chain Reaction (RT-PCR)

Ca_v_ channel subtype and auxiliary subunits mRNA expression profiles were investigated in SH-SY5Y cells using standard RT-PCR and specific primers. The primers were designed using The Basic Local Alignment Search Tool (BLAST) [60], [61], or otherwise specified as, previously described in the literature. Primer sequences, Gene Bank reference numbers, predicted PCR product sizes, and optimum annealing temperatures are shown in **Table 1**. The primers used to identify Ca_v_ subtypes and auxiliary subunits were designed so that all splice variants of specific isoforms would be amplified. On the other hand, primers to amplify Ca_v_2.2 splice variants isoform were designed to be specific to each isoform. PCR conditions to detect splice variants were set as previously described [16], [17], with gradient PCR performed for all sets of primers, allowing the identification of optimal annealing temperatures. Different sets of primers were used to identify the full length and isoforms Δ_1_ and Δ_2_ (see table 1) [16]. These primers were designed based on the region of the domain II-III linker of Ca_v_2.2 channels, as previously described [16]. Primers used to identify the full length α_1B1_ and short α_1B2_ isoforms were designed based on the C-terminus region [17]. Data is representative of at least three independent experiments.

SH-SY5Y cells (1×10^6^) were harvested and total RNA isolated using Trizol® Reagent (Invitrogen, Carlsbad, CA). The isolated RNA was subsequently treated with RNase-free DNase to remove any genomic DNA contamination. RNA concentration was determined by absorbance measurements at 260 nm and its purity/integrity was accessed by analyzing the ratio 260/280 nm with a Nanodrop® (Thermo Scientific). Synthesis of first strand cDNA was performed using 1 µg of the extracted RNA and the Omniscript Reverse Transcription Kit (Qiagen), according to the manufacturer’s instructions. cDNA amplifications were performed using Taq Polymerase (New England Biolabs, US). The reaction mix (total 25 µL) included (µL): 1 cDNA (100 ng), 0.125 of the enzyme, 0.5 reverse and 0.5 forward primers (10 µM), 0.5 dNTPs (10 mM), 2.5 Thermopol reaction buffer (10×) and nuclease free water. RT-PCR was carried through as an initial denaturation step at 95°C for 3 min followed by 35 cycles of the steps: 95°C for 30 s, optimal annealing temperature as previously determined (**Table 1**) for 60 s, 68°C extension for 60 s, plus an extra 5 min elongation step at 68°C. PCR products were analyzed by 1% agarose gel and predicted sizes estimated by comparison with DNA molecular weight makers (50 and 100 bp ladder, New England Biolabs). Target-specific primers for the housekeeping gene GAPDH were designed as previously described [19]. PCR master mix using random primers without cDNA was used as negative gDNA control in all PCRs. Specificity of primers was demonstrated in a range of control experiments (data not shown), including detection of Ca_v_2.2 plasmid but no other Ca_v_ subtypes by Ca_v_2.2 primers; and absence of detectable levels of Ca_v_2.2 in HEK cells. β_1_ and α_2_δ_1_ primers were positive for β_1_ and α_2_δ_1_ plasmids, while the same primers were negative for β_2–4_ and α_2_δ_2–3_ (data not shown), indicating primers were selective for β_1_ and α_2_δ_1_ auxiliary subunits. In addition, identity of PCR products was further confirmed by sequencing analysis (data not shown). Figures 1A–D is representative of the average of 3–10 individual experiments.

### Sequencing

PCR amplicons were first separated on agarose gels and bands of expected sizes identified. PCR products were purified using the Wizard SV Gel and PCR clean-up system (Promega), and a sample of each purified PCR product was sent for sequencing at the Australian Genome Research Facility. cDNA sequences of human Ca_v_ subtypes and auxiliary subunits were retrieved from GenBank (http://www.ncbi.nlm.nih.gov/Entrez/) and BLASTn [62] was used for confirmation of the identity of human Ca_v_ subtypes and auxiliary subunits.

### Cell Culture

The human neuroblastoma SH-SY5Y cells (Victor Diaz, Goettingen, Germany) were cultured and routinely maintained at 37°C and 5% CO_2_ in RPMI 1640 antibiotic-free medium (Invitrogen) supplemented with 10% heat-inactivated FBS and 2 mM GlutaMAX™ (Invitrogen). Trypsin/EDTA was used to detach the cells from the T-75 or T175 flasks and cells were split in a ratio of 1∶5–1∶10 every 3–4 days or when ∼80% confluent.

### Membrane Preparation for the Radioligand Binding Assay

Radioligand binding assays were performed using rat brain or SH-SY5Y cell membranes prepared as described by Wagner, *et al*., 1988 [63] with slight modification. For rat brain membranes, male Wistar rats weighing 175–250 g were sacrificed by cervical dislocation and the whole brain was rapidly removed and dissected on ice. At 4°C, tissue was re-suspended in 50 mM HEPES, pH 7.4 (50 mg wet weight tissue/ml buffer), homogenized using a Brinkmann Polytron homogenizer and centrifuged for 15 min at 40,000×g. The pellet was re-suspended in 50 mM HEPES and 10 mM EDTA at pH 7.4, incubated on ice for 30 min and centrifuged at 40,000×g for 10 min. The pellet was then re-suspended in 50 mM HEPES pH 7.4 containing 10% glycerol, aliquots were made and kept at –80°C prior to use. Bicinchoninic acid (BCA) assay reagent (Pierce Rockford, IL) was used for protein quantification.

SH-SY5Y cell membranes were harvested using trypsin/EDTA, washed once with DPBS, and centrifuged for 4 min at 500×g. After centrifugation, the supernatant was discarded and the pellet re-suspended in 10 ml binding assay buffer at pH 7.2 containing (mM): 20 HEPES, 75 NaCl, 0.2 EDTA, 0.2 EGTA and complete protease inhibitor (Roche Diagnostics, AU) and sonicated. The homogenates were then centrifuged for 30 min at 40,000×g and 4°C. The supernatant was discarded and the pellet dissolved in aliquots of binding assay buffer containing 10% glycerol stored at –80°C prior to use. BCA was used for protein quantification.

### Whole Cell Preparation for the Radioligand Binding Assay

Whole cells were prepared as described for SH-SY5Y cell membranes with the following modifications: after cells were harvested and centrifuged, the supernatant was discarded and the pellet re-suspended in sufficient volume of binding buffer to plate 50 µL/well in triplicates in 96 well plates. Specific ω-conotoxins binding was determined using the same concentration of protein as used for SH-SY5Y cell membranes (20 µg/50 µL), corresponds to 600.000 cells per well.

### Radioligand Binding Assay

Tyr22-[^125^I]-GVIA, was prepared using IODOGEN, as previously described by Ahmad [64], purified using reverse phase HPLC and stored at 4°C for use within 3 weeks. On the day of the assay, membranes were thawed on ice and reconstituted to 10 µg/50 µL (rat) or 10–20 µg/50 µL (SH-SY5Y) in binding assay buffer containing 2% complete protease inhibitor and 0.1% bovine serum albumin. Stock [^125^I]-GVIA was diluted to 20000 cpm/50 µL or 30 pM. For displacement studies, [^125^I]-GVIA was incubated with rat brain or SH-SY5Y membranes or whole cells and varying concentrations of the competing ligand in triplicates in 96 well plate formats. The plates were incubated with shaking for 1 h at room temperature and vacuum filtered through a glass fiber filter pre-soaked in 0.6% polyethyleneimine (PEI), to reduce non-specific binding and washed with buffer containing (mM) 20 HEPES and 125 NaCl at pH 7.2 using a vacuum system (Tomtec harvester). The filters were then dried at 37°C before being placed in sample bags and soaked in liquid scintillant. Radioactivity was counted using a Microbeta Jet (Wallac, Finland). The non-specific binding was determined in the presence of 50 µL of unlabeled peptides.

### Intracellular Ca^2+^ Response Measurement Using the FLIPR

SH-SY5Y cells were seeded onto 96-well or 384-well flat, clear bottom, black-walled imaging plates (Corning, Lowell, MA, US) at a density of 160,000 or 40,000 cells/well, respectively, resulting in 90–95% confluent monolayer after 48 h. On the day of the Ca^2+^ imaging assays, cells were loaded for 30 min in the dark at 37°C with 5 µM Fluo-4 acetomethoxyester (Fluo-4-AM), in physiological salt solution (PSS composition: NaCl 140 mM, glucose 11.5 mM, KCl 5.9 mM, MgCl_2_ 1.4 mM, NaH_2_PO_4_ 1.2 mM, NaHCO_3_ 5 mM, CaCl_2_ 1.8 mM, HEPES 10 mM, pH 7.4) containing in addition 0.3% BSA and 10 µM nifedipine. After the incubation period, the cells were washed once with 100 µL assay buffer (no Fluo-4-AM or BSA), and replaced with 100 µL of the same buffer. Plates were then transferred to the FLIPR^TETRA^ (Molecular Devices, Sunnyvale, CA) fluorescent plate image reader, camera gain and intensity were adjusted for each plate to yield between 800–1000 arbitrary fluorescence units (AFU) baseline fluorescence, and Ca^2+^ responses measured using a cooled CCD camera with excitation at 470–495 nM, and emission at 515–575 nM. Ten baseline fluorescence readings were taken prior to the addition of antagonists, and then fluorescent readings every 2 s for 300 s before 90 mM KCl/5 mM CaCl_2_ buffer was added and fluorescence readings again recorded each second for further 300 s. To ensure full inhibition of Ca_v_1 responses, the cells were pre-incubated for 40 min with 10 µM nifedipine. To ensure full inhibition of Ca_v_2.2 responses, the cells were pre-incubated for 10 min with 1–3 µM CVID.

### Statistical Analysis

Concentration-response curves were determined following nonlinear regression analysis using a 4-parameter Hill equation, with variable Hill slope fit to the functional assays data and one site fit to the radioligand binding assays; and normalized using GraphPad Prism (Version 5.00, San Diego, California). Negative and positive controls (PSS buffer and KCl 90 mM +5 mM CaCl_2_, respectively) were used to normalize functional data. All data is presented as mean ± SEM of 6–10 independent experiments performed in triplicate, unless otherwise stated. Statistical significance was determined using analysis of variance (ANOVA) or student’s t-test, with statistical significance defined as *p*<0.05, unless otherwise stated.
